# Multimodal Supervised Exercise Training Is Effective in Improving Long Term Walking Performance in Patients with Symptomatic Lower Extremity Peripheral Artery Disease

**DOI:** 10.3390/jcm10102057

**Published:** 2021-05-11

**Authors:** Barbara Ney, Stefano Lanzi, Luca Calanca, Lucia Mazzolai

**Affiliations:** Division of Angiology, Heart and Vessel Department, Lausanne University Hospital, 1011 Lausanne, Switzerland; stefano.lanzi@chuv.ch (S.L.); luca.calanca@chuv.ch (L.C.); lucia.mazzolai@chuv.ch (L.M.)

**Keywords:** walking capacities, 6 min walking test, long-term effects, vascular rehabilitation

## Abstract

This study aimed to evaluate the effect of a multimodal supervised exercise training (SET) program on walking performance for 12 months in patients with symptomatic lower extremity peripheral artery disease (PAD). Consecutive patients with Fontaine stage II PAD participating in the SET program of our hospital were retrospectively investigated. Walking performance, assessed using a treadmill with measures of the pain-free and maximal walking distance (PFWD, MWD, respectively), and 6 min walking distance (6MWD), were tested before and following SET, as well as at 6 and 12 months after SET completion. Ninety-three symptomatic patients with PAD (65.0 ± 1.1 y) were included in the study. Following SET, the walking performance significantly improved (PFWD: +145%, *p* ≤ 0.001; MWD: +97%, *p* ≤ 0.001; 6MWD: +15%, *p* ≤ 0.001). At 6 months, PFWD (+257%, *p* ≤ 0.001), MWD (+132%, *p* ≤ 0.001), and 6MWD (+11%, *p* ≤ 0.001) remained significantly improved compared with the pre-SET condition. At 12 months, PFWD (+272%, *p* ≤ 0.001), MWD (+130%, *p* ≤ 0.001), and 6MWD (+11%, *p* ≤ 0.001) remained significantly improved compared with the pre-training condition. The walking performance remained significantly improved in both women and men for up to 12 months (*p* ≤ 0.001). Multimodal SET is effective at improving walking performance in symptomatic patients with PAD, with improvements lasting up to 12 months.

## 1. Introduction

Lower extremity peripheral artery disease (PAD) affects over 200 million men and women worldwide [1]. Prevalence increases with age, affecting 20% of the population over 70 years old, and will become more and more frequent with the current aging of the population. PAD is mostly secondary to atherosclerotic arterial narrowing, leading to considerable impairment in walking performance and quality of life. Patients with PAD have substantially lower physical activity levels compared with people without PAD [2,3]. Symptomatic PAD manifests with intermittent claudication (IC) in as much as 50% of patients. Both symptomatic and asymptomatic patients with PAD experience greater functional impairment, faster functional decline, and higher rates of mobility loss than people without PAD [3,4,5]. Therefore, it is of great importance to improve walking performance and physical function, and to prevent mobility loss in this population. The best medical therapy includes strict control of cardiovascular risk factors, a healthy lifestyle, and exercise [2,3,4,5,6].

Supervised exercise training (SET) is recommended as a first-line intervention in symptomatic patients with PAD [2,3,4,5,6]. Several studies have shown significant walking performance improvements following SET, even in comparison with endovascular revascularisation [7,8]. Although positive effects of SET are well documented [9,10], few studies have investigated the persistence of improvement over time [11,12,13]. Moreover, the majority of these studies have not reported gender-specific analyses, even though a faster functional decline with greater mobility loss, poorer outcomes after lower extremity revascularization procedures, and a two-fold higher mortality rate with symptomatic disease were found in women with PAD compared with men with PAD [14,15].

Therefore, the first aim of this study was to assess whether the benefits of SET persisted long-term following the completion of the program in symptomatic patients with PAD. Secondly, we evaluated gender influence in long-term walking performance following SET.

## 2. Methods

### 2.1. Study Population

Consecutive symptomatic patients with chronic stage II (Fontaine’s classification) PAD participating in the multimodal SET program at the Division of Angiology of the Lausanne University Hospital (CHUV), between 2011 and 2016, were retrospectively investigated. The study was approved by the local ethics committee.

### 2.2. Multimodal Supervised Exercise Training Program

The multimodal SET consisted of three 50-min sessions per week over a 3-month period (for a total of 36 sessions) and was coordinated by a vascular physician, and all sessions were supervised by a clinical exercise physiologist specialist in vascular rehabilitation [16,17].

One third of the sessions were performed indoors, with exercises focusing on lower limb strengthening, aiming at improving muscular strength and balance. Specific functional walking (toe, skipping, side-to-side, backward, and power-jogger walking), coordination, and balance exercises were proposed. In addition, resistance exercises focusing on main lower limb muscles (squat, calf/heel raises, and lunges) using body weight or elastic bands were also proposed. At the beginning of the SET, patients were asked to perform 5–15 repetitions of each resistance exercise using their body weight, interspersed with 30 to 60 s of recovery. Subsequently, if tolerated, patients were encouraged to perform 20–30 repetitions of each resistance exercise using body weight, or 10–20 repetitions using the elastic band. Training intensity was mainly set at 10–14 on the 6–20 Borg scale (low-to-moderate intensity) [18].

The other two thirds of the exercise training time were performed outdoors (if the weather allowed) with Nordic walking. The patients were taught the proper way of walking with the poles and were asked to walk until reaching a level of moderate-to-severe claudication, and then paused until complete resolution of the pain, before resuming walking [19]. During Nordic walking sessions, training intensity was mainly set at 12–14 on the 6–20 Borg scale (moderate intensity). During each Nordic walking session, the number of steps were recorded using a pedometer (Tanita PD-725). Attendance rate to SET was defined as the percentage of attended sessions out of the total number of sessions. During this 3-month period, patients also received structured information in the form of four 90-min educational workshops about PAD, physical activity, and risk factors concerning nutrition and tobacco use.

At the end of the program, patients were encouraged to keep exercising on a regular basis, but they were not contacted and were only seen at the 6- and 12-month follow-ups.

### 2.3. Measures

For each patient, the medical history was taken, and a physical examination, including thorough vascular evaluation, was performed. Our SET program routinely assesses cardiac contraindication to exercise by cardiac ergometry or cardiac MRI, when necessary.

Vascular parameters were assessed at each endpoint (that is, prior to and at the end of SET, as well as 6 and 12 months following SET). The ankle-brachial index (ABI; ratio of the highest ankle systolic blood pressure of each limb to the highest humeral systolic pressure) was measured at rest and immediately after the treadmill test using a hand-held 5-MHz Doppler flow meter (Atys Medical, France). The toe-brachial index (TBI; ratio of toe pressure to the highest humeral pressure) was measured at rest and immediately following the treadmill test by photoplethysmography (Atys Medical, France). Transcutaneous oxygen pressure (TcPO_2_) was measured both in the sitting and supine positions using a TCM4 Radiometer (Medical Aps, Denmark) with the electrode positioned on the forefoot.

Each patient underwent a thorough ultrasound (Philips HD15) vascular examination of the lower limbs to determine the level and severity of stenosis or occlusion.

A constant-load treadmill test was also performed at each endpoint to determine the pain-free and maximal walking distance (PFWD and MWD, respectively). PFWD was defined as the distance in meters until the onset of pain in the lower limbs, and MWD was defined as the distance until patients reached maximum claudication and stopped walking [19]. The treadmill test was performed at 3.2 km/h speed with a 12% slope. In some cases, the speed was adapted depending on the patient’s safety and exercise tolerance. In this case, the same parameters were used throughout the experimental period.

A six-minute walking test was also performed at each endpoint to determine the six-minute walking distance (6MWD). Patients were asked to walk as far as possible along an indoor 50 m corridor [20,21]. The six-minute walking test was always performed in the same corridor and was assessed by the same operator, but was performed during a dedicated session, distinct from the vascular and treadmill assessment visits.

### 2.4. Statistical Analysis

Data were analyzed with an intention-to-treat approach. To that end, multiple imputations were performed for patients who dropped out during the SET program and for patients who did not attend a complete evaluation at all endpoints. A fully conditional specification with predictive mean matching, to impute all variables simultaneously, was used. Multiple imputations for missing data were performed to obtain twenty imputed data sets. Age, gender, vascular parameters, walking performance (treadmill and six-minute walk test), cardiovascular risk factors, ongoing treatment, and comorbidities were used to impute the data sets. Chi-square and *T*-tests were used to compare the baseline characteristics of patients who completed the evaluation at all endpoints vs. those who dropped out during the SET program and/or did not attend a complete evaluation at all endpoints. In addition, chi-square and *T*-tests were used to compare the baseline characteristics of the gender subgroup analyses (men and women). For all of the variables, the normality of distribution was visually and statistically assessed (Kolmogorov–Smirnov test). When the normality assumption was not met, data were log-transformed. A one-way repeated measures analysis of variance (ANOVA) was performed to compare the PFWD, MWD, 6MWD, and vascular parameters between the different endpoints (pre-, post-, 6 months, and 12 months). A two-way repeated measures ANOVA (time (pre- vs. post- vs. 6 months vs. 12 months) x group (men vs. women)) was used for a comparison of the walking performance and vascular parameters between women and men. When ANOVA (one-way and two-way) revealed a significant time effect or time x group interaction effect, significance was determined using multiple comparisons with Bonferroni adjustment. ANOVAs were adjusted for covariates (age and gender) when appropriate. All of the statistical analyses were performed with SPSS 26 software (IBM Corporation, Armonk, NY, USA). The level of significance was set at *p* ≤ 0.05.

## 3. Results

### 3.1. Study Population

Of the ninety-three patients who started the SET program, eight dropped out. Two patients underwent endovascular revascularization during the program because of worsening of claudication symptoms, and six dropped out for health reasons not related to SET (*n* = 5) and for work commitments (*n* = 1). Therefore, 85 patients (91%) attended and completed the SET program between 2011 and 2016. Of those, 70 patients attended the follow-up at 6 months, and 56 patients attended the follow-up at 12 months. The baseline characteristics of the patients who completed the evaluation at all endpoints were similar to those who dropped out during the SET program and/or who did not attend a complete evaluation at all endpoints, except for TBI, sitting TcPO_2_, and PFWD (data not shown). Patients’ general characteristics are described in Table 1.

### 3.2. Training Characteristics

The attendance rate to SET was 90%. Patients spent an average of 21 sessions (63%) of training performing Nordic walking and 11 sessions (37%) performing strengthening of lower limbs.

### 3.3. Walking Performance

Compared with pre-SET, PFWD and MWD were significantly greater at the end of SET (PFWD +145%, *p* ≤ 0.001; MWD +97%, *p* ≤ 0.001), at 6 months (PFWD +257%, *p* ≤ 0.001; MWD +132%, *p* ≤ 0.001), and at 12 months (PFWD +272%, *p* ≤ 0.001; MWD +130%, *p* ≤ 0.001; Table 2). Compared with post-SET, PFWD was significantly greater at the 6-month (+45%, *p* = 0.006) and 12-month (+51%, *p* ≤ 0.001) follow-up. Compared with the post-SET, there were no significant changes in MWD at the 6- and 12-month follow-up. There were no significant changes between 6 and 12 months for the PFWD and MWD (Table 2).

Compared with the pre-SET, 6MWD was significantly greater at the end of SET (+15%, *p* ≤ 0.001), at 6 months (+11%, *p* ≤ 0.001), and at 12 months (+11%, *p* ≤ 0.001; Table 2). Compared with post-SET, there were no significant changes in 6MWD at the 6- and 12-month follow-up. There were no significant changes between the 6- and 12-month follow-ups for 6MWD (Table 2).

### 3.4. Walking Performance and Gender Subgroup Analysis

The baseline characteristics of men and women were similar and are presented in Table 3. The two-way ANOVA revealed that following SET, PFWD, MWD, and 6MWD similarly increased in both women and men (significant time effect with no significant group and time x group interaction effect), and that improvements were maintained up to 12 months (Table 4).

Multiple comparisons analysis for time effect revealed that, compared with pre-SET, PFWD was significantly greater at the end of SET (women +230%; men +108%; *p* ≤ 0.001), at 6 months (women +254%; men +258%; *p* ≤ 0.001), and at 12 months (women +326%; men +247%; *p* ≤ 0.001; Table 4). In addition, multiple comparisons analysis for time effect revealed that, compared with post-SET, PFWD was significantly greater at 6 months (women +7%; men +72%; *p* = 0.006), and at 12 months (women +29%; men +67%; *p* ≤ 0.001; Table 4). There were no significant changes between 6 and 12 months for the PFWD (Table 4).

Multiple comparisons analysis for time effect revealed that, compared with pre-SET, MWD was significantly greater at the end of the SET (women +121%; men +87%; *p* ≤ 0.001), at 6 months (women +134%; men +130%; *p* ≤ 0.001), and at 12 months (women +127%; men +131%; *p* ≤ 0.001; Table 4). There were no significant changes between post-SET and 6 months, and also between 6 and 12 months for the MWD (Table 4).

Multiple comparisons analysis for time effect revealed that, compared with pre-SET, 6MWD was significantly greater at the end of SET (women +19%; men +13%; *p* ≤ 0.001), at 6 months (women +14%; men: +10%; *p* ≤ 0.001), and at 12 months (women +12%; men +10%; *p* ≤ 0.001; Table 4). There were no significant changes between post-SET and 6 months, and also between 6 and 12 months for the MWD (Table 4).

### 3.5. Vascular Parameters

One-way ANOVA revealed significant changes for ABI over time (Table 5). Multiple comparisons analysis revealed that ABI was significantly higher at 6-month follow-up compared with post-SET (*p* = 0.006; Table 5).

One-way ANOVA revealed significant changes for TBI over time (Table 5). Multiple comparisons analysis revealed that, compared with pre-SET, TBI was significantly greater at the end of the SET (*p* = 0.009), at 6-month (*p* ≤ 0.001), and at 12-month (*p* ≤ 0.001) follow-ups (Table 5). In addition, compared with post-SET, TBI was significantly greater at the 12-month follow-up (*p* = 0.037; Table 5).

One-way ANOVA revealed significant changes for supine and sitting TcPO_2_ over time (Table 5). Multiple comparisons analysis revealed that supine and sitting TcPO_2_ at the 12-month follow-up were significantly greater than the pre-SET (*p* ≤ 0.001; Table 5) and the 6-month follow-up (*p* = 0.002; Table 5).

### 3.6. Vascular Parameters and Gender Subgroup Analysis

Two-way ANOVA revealed that no significant changes were observed for ABI over time for both men and women (no significant time, group, and time × group interaction effect; Table 6).

Two-way ANOVA revealed significant changes for TBI over time (Table 6). Multiple comparisons analysis for time effect revealed that, compared with pre-SET, TBI was significantly greater at 6 (*p* = 0.028) and 12 months (*p* = 0.002; Table 6). There were no significant changes between pre-SET and post-SET, post-SET and 6 months, and also between 6 and 12 months for the TBI (Table 6). There was no significant group or time x group interaction effect for the TBI (Table 6).

Two-way ANOVA revealed significant changes for TcPO_2_ (supine and sitting) over time (Table 6). Multiple comparisons analysis for time effect revealed that supine TcPO_2_ at 12 months was significantly greater than pre-SET (*p* = 0.011) and 6 months (*p* = 0.001; Table 6). Multiple comparisons analysis for time effect revealed that sitting TcPO_2_ at 12 months was significantly greater than the pre-SET (*p* = 0.002) and 6 months (*p* ≤ 0.001; Table 6). There was no significant time x group interaction effect, whereas a significant group effect was observed for TcPO_2_ (supine and sitting) showing greater values in men compared with women (Table 6).

## 4. Discussion

The results presented herein show the following: (1) a persistence of significant walking performance improvement (PFWD, MWD, and 6MWD) at 6 and 12 months, and (2) that women benefited as much as men from SET.

Past studies have abundantly proved the effectiveness of SET on walking performance in patients with claudication [8,11,12,13,16,22,23,24]. In agreement with these studies, our results show an improvement in walking capacities assessed by PFWD, MWD, and by 6MWD after a 3-month SET.

Most of the earlier trials with long-term follow-ups from 12 months to 7 years used treadmill or resistance training, with outcomes measured using treadmill testing and/or quality of life questionnaires. Nevertheless, all tended to confirm the persistence of an improvement of walking capacity after SET [7,8,11,12,13,22,23,25]. Keo et al. [11] published, in 2008, a prospective study of 40 patients with a follow-up of 39 ± 20 months after a 3-month SET. The results showed a sustainable improvement of PFWD and MWD at follow-up, but 6MWD was not tested in this study [11]. In line with these results, our study shows a persistence of improvement for walking distances assessed by treadmill testing, for up to 12 months following SET. In addition, compared with the post-SET condition, PFWD further increased at the 6- and 12-month follow-ups. On the other hand, controversies exist as to whether SET programs also elicit functional (that is, 6MWD) long-term benefits. The results of this present study showed the persistence of a significant improvement in 6MWD over time when compared with the pre-SET condition. Moreover, although slightly decreased, when compared with the post-SET condition, 6MWD remained unchanged at the 6- and 12-month follow-ups. These different evolutions over time in the treadmill performance and 6MWD confirm that these different outcomes are not completely interchangeable to measure walking capacities and endurance in patients with PAD [20,26]. Indeed, the improvement in walking distances assessed using a treadmill could be influenced by the learning effect generated from the repetition of tests, whereas the six-minute walk test is not associated with such a learning effect [20,26]. In addition, 6MWD is more representative of daily life walking [27], and is more correlated with daily physical activity than treadmill testing [20,26,27]. Contrary to the maximal treadmill test, changes in 6MWD have been related to clinically meaningful outcomes, such as mortality and mobility loss in patients with PAD [28]. Our results showed sustainability of functional walking endurance up to 12 months following multimodal SET.

McDermott et al. [25] recently showed no persistent improvements in six-minute walking distance 6 months after SET completion in symptomatic and asymptomatic patients with PAD, trained by treadmill exercise or supervised resistance training. The authors stated that these results may be related to a decline in physical activity levels and/or training modality of participants in that trial [25]. Indeed, treadmill exercise training may improve treadmill performance more than 6MWD, because of the learning effect [20,25], and this treadmill learning effect is probably not transferable to the six-minute walking test. Contrary to these results [25], the findings of the present investigation showed functional long-term effects of multimodal SET in patients with PAD. Although the design of this study cannot discern whether multimodal SET is superior to treadmill training in improving functional outcomes over time, our results may be related to the functional characteristic of the training modalities used in our study. Indeed, participants in the present study performed a functional (overground) multimodal SET program combining Nordic walking and the strengthening of lower limbs. This type of training is well correlated to everyday efforts and may allow patients to carry on with their training even after completion of SET. Further studies are needed in order to better assess this speculation in the future. Interestingly, our results are in line with those of McDermott et al. [29], who previously showed that patients with PAD randomized in a group-mediated cognitive behavioral intervention aimed to promote a functional home-based exercise, but also to achieve long-term lifestyle adherence (GOALS trial), still had a greater 6MWD after a 6-month follow-up. Taken together, these results highlight the pivotal role of functional exercise in maintaining long-term benefits in patients with PAD.

The majority of PAD studies did not report gender-specific analyses or outcomes [30], even though women suffer the consequences of PAD at rates at least as high as those observed in men. Women with PAD have greater functional impairment [4] and a more rapid functional decline than women without PAD [14]. Gardner et al. showed that women present 33% shorter PFWD and 23% shorter MWD than men, concluding that women should receive priority for participating in exercise rehabilitation programs [31]. In 2015, Gommans et al. showed a significantly lower MWD in women than in men during the first 3 months of SET and after 1-year follow-up [32]. The results of the present investigation are in contrast with previous findings. First, we observed that women presented greater (although not significant) baseline treadmill walking performance (PFWD and MWD). On the other hand, women had lower (although not significantly) baseline 6MWD values. Second, we observed similar benefits in women and men following multimodal SET in patients with PAD. Although our results showed no group and no time x group interaction effect for walking performance, suggesting a similar improvement in men and women following SET and over the different endpoints, it is interesting to note that women have greater improvements (that is, delta) than men. The present study cannot explain the mechanisms underlying these findings, but signifies the need for future studies investigating the effect of SET on women. Even though the importance of screening and treating women for atherosclerotic diseases is now widely acknowledged, our findings support the fact that women should equally be encouraged to participate in SET programs [32,33,34,35].

As expected, ABI did not change between pre- and post-SET, which is in line with recent findings [10]. However, in our study, we observed that ABI was significantly increased at 6 months compared with the post SET values (mean difference: 0.06). In addition, as we already showed elsewhere [16], TBI increased significantly following SET (mean difference 0.04). Surprisingly, the values remained significantly higher at each following endpoint. Compared with before SET, TcPO_2_ was significantly increased at 12 months (mean difference 5.5 mmHg for supine and 5.3 mmHg for sitting TcPO_2_). Interestingly, similar results were obtained in women and men in the gender subgroup analyses. The clinical relevance of these changes remains questionable. ABI and TBI have been described to have a heterogeneous reliability, affected by co-morbidities such as medial arterial calcification and diabetes [36,37]. Therefore, we consider that our results, even if statistically significant, have no clinical repercussions.

Our results clearly demonstrate that SET remains a pillar of PAD treatment. Exercise is also beneficial for other manifestation of atherosclerotic disease, such as coronaropathy or cerebrovascular disease, as well as for many other health conditions [38,39,40], and should therefore be encouraged in all patients with PAD. Guidelines from the European Society for Vascular Medicine and the American Heart Association recommend SET whenever available with a class of recommendation of either I B and I A, respectively [2,6,19,41,42]. The main obstacle for implementing SET as routine first line management is the lack of regional availability and patient’s motivation [43,44]. For this reason, vascular specialists as well as any other professionals treating patients with PAD should strongly encourage exercise training as an inherent part of treatment for claudication [45].

Some methodological considerations exist and need to be addressed. First, the non-randomized and retrospective character of the study. Second, missing data had to be replaced using multiple imputations. However, the latter procedure may have avoided a possible selection bias (that is, only patients who were able/volunteer to attend all endpoint assessment were included) and allowed for increased statistical power. Third, the study lacked a control group. The lasting beneficial effect of multimodal SET should be confirmed by a prospective randomized control trial, which should include a control group without exercise intervention and/or different training groups performing other training modalities. This should discriminate which training modality has better (long-term) impacts on walking performance and physical function. Fourth, a change in baseline characteristics (such as smoking or medications) or information about home exercise regimen during the follow up were not collected, which may have impacted walking distance and, therefore, outcomes of the study.

In conclusion, the results showed that multimodal SET is effective at improving walking performance and physical function in men and women with symptomatic PAD, and that the benefits last up to 12 months of follow-up.

## Figures and Tables

**Table 1:** Characteristics of patients.

Number of Included Patients	93
Age, y (mean ± SE)	65.0 ± 1.1
BMI, kg/m^2^ (mean ± SE)	27.3 ± 0.5
Women, *n* (%)	26 (28)
**Cardio-vascular Risk Factors**	
Type 2 diabetes mellitus, *n* (%)	32 (34)
Hypertension, *n* (%)	67 (72)
Smoking (current), *n* (%)	45 (48)
Smoking (former), *n* (%)	38 (41)
Smoking (never), *n* (%)	10 (11)
Hypercholesterolemia, *n* (%)	72 (77)
Family history of CVD, *n* (%)	18 (19)
**Prior History of CVD**	
Cardiac, *n* (%)	26 (28)
Cerebrovascular, *n* (%)	5 (5)
**Co-morbidities**	
Orthopaedic, *n* (%)	35 (38)
Cardiac disease, *n* (%)	26 (28)
Kidney disease, *n* (%)	12 (13)
Lung disease, *n* (%)	14 (15)
Other, *n* (%)	16 (17)
**Stenosis Localization**	
Aorto-iliac, *n* (%)	17 (18)
Ilio-femoral, *n* (%)	3 (3)
Femoro-popliteal, *n* (%)	63 (68)
Distal, *n* (%)	5 (5)
Proximal-distal, *n* (%)	5 (5)
Prior arterial revascularization, *n* (%)	54 (58)
**Ongoing Treatment**	
Antiagregant, *n* (%)	84 (90)
Antihypertensive, *n* (%)	65 (70)
Lipid lowering, *n* (%)	67 (72)
Anticoagulant, *n* (%)	12 (13)
Antidiabetic, *n* (%)	31 (33)

SE—standard error; BMI—body mass index; CVD—cardio-vascular disease.

**Table 2:** Evolution of PFWD, MWD, and 6MWD after supervised exercise training (SET).

	Pre	Post	6-Month	12-Month	*p* Value
PFWD (m) ^a^	93.8 ± 7.9	230.1 ± 28.5 *	334.5 ± 31.9 *^,£^	348.5 ± 30.7 *^,#^	≤0.001
MWD (m) ^a^	284.5 ± 23.5	561.7 ± 46.5 *	658.8 ± 40.2 *	653.6 ± 37.2 *	≤0.001
6MWD (m)	410.6 ± 9.0	472.1 ± 9.7 *	455.3 ± 7.2 *	454.3 ± 6.2 *	≤0.001

Values are presented as the mean ± SE. PFWD—pain free walking distance; MWD—maximal walking distance; 6MWD—six-minute walking distance. * *p* ≤ 0.001 for significant difference compared to pre-SET condition (multiple comparisons with Bonferroni adjustment). ^#^ *p* ≤ 0.001 for significance compared to the post-SET condition (multiple comparisons with Bonferroni adjustment). ^£^ *p* = 0.006 for significance compared to the post-SET condition (multiple comparisons with Bonferroni adjustment). ^a^ adjusted for gender.

**Table 3:** Baseline characteristics of men and women.

	Women	Men	*p*-Value
Age, y (mean ± SE)	64.5 ± 2.3	65.2 ± 1.2	0.784
BMI, kg/m^2^ (mean ± SE)	26.6 ± 1.1	27.6 ± 0.6	0.398
**Walking Performance**			
PFWD, m (mean ± SE)	103.6 ± 18.4	89.9 ± 8.3	0.503
MWD, m (mean ± SE)	313.0 ± 49.2	273.4 ± 26.5	0.483
6MWD, m (mean ± SE)	403.7 ± 19.2	413.3 ± 10.2	0.660
**Vascular Parameters**			
ABI (mean ± SE)	0.90 ± 0.05	0.79 ± 0.03	0.067
TBI (mean ± SE)	0.64 ± 0.04	0.57 ± 0.02	0.109
TcPO_2_ supine, mmHg (mean ± SE)	48.7 ± 2.0	47.8 ± 1.5	0.715
TcPO_2_ sitting, mmHg (mean ± SE)	56.8 ± 2.2	59.5 ± 1.3	0.302
**Cardio-Vascular Risk Factors**			
Type 2 diabetes mellitus, *n* (%)	5 (19)	27 (40)	0.055
Hypertension, *n* (%)	18 (69)	49 (73)	0.707
Smoking (current), *n* (%)	15 (58)	30 (45)	0.263
Smoking (former), *n* (%)	8 (31)	30 (45)	0.218
Smoking (never), *n* (%)	3 (11)	7 (10)	0.879
Hypercholesterolemia, *n* (%)	21 (81)	51 (76)	0.630
Family history of CVD, *n* (%)	5 (19)	13 (19)	0.985
**Prior History of CVD**			
Cardiac, *n* (%)	7 (27)	19 (28)	0.890
Cerebrovascular, *n* (%)	1 (4)	4 (6)	0.684
**Co-morbidities**			
Orthopaedic, *n* (%)	14 (54)	21 (31)	0.044
Cardiac disease, *n* (%)	6 (23)	20 (30)	0.514
Kidney disease, *n* (%)	4 (15)	8 (12)	0.657
Lung disease, *n* (%)	5 (19)	9 (13)	0.483
Other, *n* (%)	6 (23)	10 (15)	0.350
Prior arterial revascularization, *n* (%)	11 (42)	43 (64)	0.055
**Ongoing Treatment**			
Antiagregant, *n* (%)	22 (85)	62 (93)	0.168
Antihypertensive, *n* (%)	17 (65)	48 (72)	0.555
Lipid lowering, *n* (%)	19 (73)	48 (72)	0.890
Anticoagulant, *n* (%)	4 (15)	8 (12)	0.657
Antidiabetic, *n* (%)	5 (19)	26 (39)	0.072

BMI—body mass index; PFWD—pain free walking distance; MWD—maximal walking distance; 6MWD—six-minute walking distance. ABI—ankle-brachial index; TBI—toe-brachial index; TcPO_2_—transcutaneous oxygen pressure; CVD—cardio-vascular disease.

**Table 4:** Walking performance in women and men before, after, 6 months, and 12 months post supervised exercise training (SET).

		Pre	Post	6-Month	12-Month	Group Effect	Time Effect	Time × Group Effect
PFWD (m)	Men	89.9 ± 8.3	186.8 ± 20.6 *	321.8 ± 35.1 *^,£^	312.3 ± 24.4 *^,#^	0.329	≤0.001	0.458
	Women	103.6 ± 18.4	341.6 ± 84.3 *	367.2 ± 70.1 *^,£^	441.7 ± 88.9 *^,#^			
MWD (m)	Men	273.4 ± 26.5	511.1 ± 48.3 *	629.8 ± 42.5 *	632.0 ± 38.6 *	0.427	≤0.001	0.529
	Women	313.0 ± 49.2	692.3 ± 107.9 *	733.6 ± 93.4 *	709.4 ± 88.7 *			
6MWD (m)	Men	413.3 ± 10.2	468.7 ± 10.9 *	453.5 ± 9.2 *	455.0 ± 7.0 *	0.999	≤0.001	0.392
	Women	403.7 ± 19.2	480.7 ± 20.3 *	460.1 ± 9.6 *	452.4 ± 12.9 *			

Values are the means ± SE. PFWD—pain free walking distance; MWD—maximal walking distance; 6MWD—six-minute walking distance. * *p* ≤ 0.001 for significant difference compared to pre-SET condition (multiple comparisons with Bonferroni adjustment). ^#^ *p* ≤ 0.001 for significance compared to the post-SET condition (multiple comparisons with Bonferroni adjustment). ^£^ *p* = 0.006 for significance compared to the post-SET condition (multiple comparisons with Bonferroni adjustment).

**Table 5:** Evolution of vascular parameters after supervised exercise training (SET).

	Pre	Post	6-Month	12-Month	*p* Value
ABI	0.82 ±0.02	0.81 ± 0.02	0.87 ± 0.02 ^#^	0.85 ± 0.02	0.014
TBI	0.59 ± 0.02	0.63 ± 0.02 ^$^	0.66 ± 0.01 *	0.68 ± 0.02 *^,¤^	≤0.001
TcPO_2_ supine (mmHg)	48.1 ± 1.2	50.7 ± 1.2	50.4 ± 0.9	53.6 ± 0.6 *^,£^	0.001
TcPO_2_ sitting (mmHg)	58.7 ± 1.1	61.1 ± 1.3	59.6 ± 1.0	64.0 ± 0.6 *^,£^	≤0.001

Values are the means ± SE. ABI—ankle-brachial index; TBI—toe-brachial index; TcPO_2_—transcutaneous oxygen pressure. * *p* ≤ 0.001 for significant difference compared to pre-SET condition (multiple comparisons with Bonferroni adjustment). ^#^ *p* = 0.006 for significant difference compared to post-SET condition (multiple comparisons with Bonferroni adjustment). ^£^ *p* = 0.002 for significant difference compared to 6-month follow-up (multiple comparisons with Bonferroni adjustment). ^$^ *p* = 0.009 for significant difference compared to pre-SET condition (multiple comparisons with Bonferroni adjustment). ^¤^ *p* = 0.037 for significant difference compared to post-SET condition (multiple comparisons with Bonferroni adjustment).

**Table 6:** Evolution of vascular parameters in women and men before, after, 6 months post, and 12 months post supervised exercise training (SET).

		Pre	Post	6-Month	12-Month	Group Effect	Time Effect	Time × Group Effect
ABI	Men	0.79 ± 0.03	0.79 ± 0.02	0.87 ± 0.02	0.82 ± 0.02	0.075	0.128	0.200
	Women	0.90 ± 0.05	0.86 ± 0.04	0.88 ± 0.04	0.90 ± 0.04			
TBI	Men	0.57 ± 0.02	0.62 ± 0.02	0.67 ± 0.02 *	0.67 ± 0.02 ^#^	0.476	≤0.001	0.058
	Women	0.64 ± 0.04	0.65 ± 0.04	0.64 ± 0.03 *	0.69 ± 0.04 ^#^			
TcPO_2_ supine (mmHg)	Men	47.8 ± 1.5	52.5 ± 1.4	51.8 ± 0.9	54.4 ± 0.7 ^$,£^	0.030	0.007	0.065
	Women	48.7 ± 2.0	46.2 ± 2.6	46.8 ± 1.9	51.8 ± 1.0 ^$^^,£^			
TcPO_2_ sitting (mmHg)	Men	59.5 ± 1.3	62.4 ± 1.5	61.2 ± 0.9	64.9 ± 0.7 ^#,¤^	0.005	0.001	0.663
	Women	56.8 ± 2.2	57.7 ± 2.5	55.4 ± 2.4	61.7 ± 1.0 ^#,¤^			

Values are the means ± SE. ABI—ankle-brachial index; TBI—toe-brachial index; TcPO_2_—transcutaneous oxygen pressure. * *p* = 0.028 for significant difference compared to pre-SET condition (multiple comparisons with Bonferroni adjustment). ^#^ *p* = 0.002 for significant difference compared to pre-SET condition (multiple comparisons with Bonferroni adjustment). ^$^ *p* = 0.011 for significant difference compared to pre-SET condition (multiple comparisons with Bonferroni adjustment). ^£^ *p* = 0.001 for significant difference compared to 6-month follow-up (multiple comparisons with Bonferroni adjustment). ^¤^ *p* ≤ 0.001 for significant difference compared to 6-month follow-up (multiple comparisons with Bonferroni adjustment).

## Data Availability

The data presented in this study are available on request from the corresponding author.
